# Characterization and a RT-RPA assay for rapid detection of Chilli Veinal mottle virus (ChiVMV) in tobacco

**DOI:** 10.1186/s12985-020-01299-w

**Published:** 2020-03-10

**Authors:** Yubing Jiao, Chuantao Xu, Jialun Li, Yong Gu, Chun Xia, Qiang Xie, Yunbo Xie, Mengnan An, Zihao Xia, Yuanhua Wu

**Affiliations:** 1 College of Plant Protection, Shenyang Agricultural University, Shenyang, 110866 China; 2 Luzhou City Company of Sichuan Tobacco Company, Luzhou, 646000 China; 3 Sichuan Province Company of China Tobacco Corporation, Chengdu, 610041 China

**Keywords:** Chilli veinal mottle virus (ChiVMV), Characterization, RT-RPA, Rapid detection, *Nicotinana tabacum*

## Abstract

**Background:**

Chilli veinal mottle virus (ChiVMV), which belongs to the genus *Potyvirus* of the family *Potyviridae*, mainly infects solanaceous plants and has caused serious economic losses in Asia and Africa. Tobacco plants infected with ChiVMV suffered from punctate necrosis of leaves, leaf deformation, systemic necrosis of leaves and stems, and eventually plant death. However, ChiVMV infection could not usually be identified given the lack of rapid and efficient detection assays in tobacco plants. Therefore, an isolate of tobacco-infecting ChiVMV (ChiVMV-LZ) was obtained, and a novel isothermal amplification and detection technique, reverse transcription-recombinase polymerase amplification (RT-RPA), was established to detect ChiVMV in tobacco plants.

**Methods:**

In this study, the full-length genome of ChiVMV-LZ was obtained using reverse transcription-polymerase chain reaction (RT-PCR) and rapid amplification of cDNA ends (RACE) assays. The genome sequence of ChiVMV-LZ was characterized by sequence alignment and phylogenetic analysis. Then, a RT-RPA assay was established for rapid and sensitive detection of ChiVMV-LZ in tobacco. Additionally, the established RT-RPA assay was compared to the RT-PCR assay in aspect of sensitivity and application in field-collected tobacco samples.

**Results:**

ChiVMV-LZ was isolated from diseased tobacco in Luzhou, Sichuan, China. The tobacco plants inoculated with ChiVMV-LZ showed typical symptoms of yellow and round spots on the leaves, and curled and folded leaf margin, similar to those observed on naturally ChiVMV-infected tobacco in the field. The full-length genomic sequence of ChiVMV-LZ was determined to be 9742 nucleotides. Sequence alignment and phylogenetic analysis showed that ChiVMV-LZ was most closely related to ChiVMV-Yp8 isolated from pepper plants in Sichuan province while distantly related to ChiVMV-YN from tobacco in Yunnan province, indicating a possibly geographical differentiation of ChiVMV isolates. Additionally, a RT-RPA assay was established for rapid detection of ChiVMV in tobacco. The RT-RPA has no cross-reaction with other related tobacco viruses and is about 10-fold more sensitive than conventional RT-PCR method.

**Conclusion:**

The characterization of ChiVMV-LZ infecting tobacco was determined, and the established RT-RPA assay provides a reliable and effective method for rapid detection of ChiVMV in tobacco.

## Background

Chilli veinal mottle virus (ChiVMV) is a positive-sense single-stranded RNA (+ssRNA) virus in the genus *Potyvirus* of the family *Potyviridae* [1]. The genomic RNA of ChiVMV contains a poly(A) tail at 3′ end and its 5´ end covalently binds to VPg protein, which is wrapped in a single type of coat protein. The open reading frame (ORF) of ChiVMV encodes a large polyprotein, which is processed into ten functional mature proteins [2, 3]. ChiVMV mainly infects solanaceous crops and has caused considerable economic losses to pepper (*Capsicum annuum* L.) production in Asia and Africa [4–12]. Moreover, it has been found highly deleterious to production of tobacco (*Nicotiana tabacum* L.) production since 2011 in China [13–15]. Tobacco plants infected with ChiVMV also present with leaf punctate necrosis or deformation, systemic necrosis of leaves and stems, and plant death finally [16, 17]. Sichuan province is the main region for tobacco production in China, where the pepper was widely dispersed around tobacco field. Therefore, the particular planting environment enables the tobacco a perfect intermediate host for ChiVMV infection and transmission. Nonetheless, the great damage from ChiVMV infection has long been overlooked in tobacco production. Recently, a survey on tobacco viral diseases showed that more than 3% of diseased tobacco plants were infected with ChiVMV in Sichuan province [18]. Given quick onset of ChiVMV and lack of acquired disease resistance for main tobacco strains, it is of great significance to further characterize ChiVMV and achieve early detection and monitoring ChiVMV infection, in order to maintain adequate tobacco production.

ChiVMV is transmitted mainly by aphids in a non-persistent manner [19]. Currently, the disease intervention for ChiVMV infection mainly relies on trapping and controlling aphid vectors. Therefore, early and effective detection methods with high sensitivity and specificity are required to prevent further spread of ChiVMV in the field. Traditional methods for ChiVMV detection consisted mainly of viral isolation and purification [20], enzyme-linked immunosorbent assay (ELISA) [21], reverse transcription-polymerase chain reaction (RT-PCR) [11] and reverse transcription loop-mediated isothermal amplification (RT-LAMP) [3]. Recently, a porous plate-format antibody array has also been used for multiplex detection of *Acidovorax citrulli*, ChiVMV, watermelon silver mottle virus (WSMoV) and melon yellow spot virus (MYSV) [22]. The commercial ELISA kits could be applied for the diagnosis of ChiVMV infection; however, they were much more expensive and less sensitive than molecular diagnostic methods. The commonly used RT-PCR was time-consuming and required expensive equipment, complex laboratory settings and experienced technician. Additionally, a relatively high temperature was needed to conducted the RT-LAMP assay, which made it difficult and impractical for ChiVMV detection in the field. Therefore, it is pivotal to establish a rapid and effective technique for on-site detection to control virus transmission and prevent disease epidemic.

Recombinase polymerase amplification (RPA) is a novel method that can isothermally amplify the nucleic acid [23]. In RPA, the recombinase forms a nucleoprotein filament with single-stranded oligonucleotide backbones, and scans the double-stranded DNA target for homologous sequences [24]. Primers for RPA are designed in a manner similar to that for PCR, which permit the establishment of an exponential amplification process [25]. At the optimal temperature (36~42 °C), the reaction proceeds rapidly and results in specific DNA amplification from a few target copies to detectable levels, which is more suitable for rapid detection of viral genomic DNA or RNA [26].

In this study, an isolate of tobacco-infecting ChiVMV (ChiVMV-LZ) was obtained and its pathogenicity was determined. The genomic sequence and phylogenetic analysis of ChiVMV-LZ were fuether analyzed to reveal its taxonomic status and relationship with other national and regional isolates. Moreover, a RT-RPA assay was established for rapid, sensitive and cost-effective detection of ChiVMV in field-grown tobacco plants.

## Methods

### Sample collection and virus detection

For the isolation of potential viral pathogens infecting tobacco plants, the symptomatic leaf samples were collected from Luzhou, Sichuan province, China in 2018. Total RNA was extracted using RNAiso Plus reagent (TaKaRa, Dalian, China) from tobacco leaves following the manufacturer’s guidelines. Viruses were detected by RT-PCR with four pairs of degenerate primers [27], including CMVCPf/CMVCPr (for cucumber mosaic virus), Tob-Uni1/Tob-Uni2 (for tobamoviruses), PotyF/PotyR (for potyviruses) and TSWVf/TSWVr (for tomato spotted wilt virus) (Additional file 1: Table S1). The amplified PCR products were purified with EasyPure Quick Gel Extraction kit (Transgen Biotech, Beijing, China), cloned into pEASY-T1 Cloning Vector (Transgen Biotech) and directly sequenced (Sangon Biotech, Shanghai, China), respectively.

### Different host reaction and detection of ChiVMV

The crude extract was obtained by homogenizing ChiVMV-infected tobacco leaf sample in phosphate buffer (0.01 M, pH 7.2) at 1:5 (w/v) ratio. The crude extract was inoculated onto five healthy plants of tobacco, pepper, tomato (*Solanum lycopersicum* L.) and alkekengi (*Physalis alkekengi* L.) by rubbing carborundum-dusted leaves according to a previous report, respectively [28]. Inoculated plants were grown under greenhouse conditions at 24~26 °C, and observed daily for symptom development. To verify the presence of ChiVMV-LZ in inoculated plants, the systemically infected leaves were harvested for RNA isolation and RT-PCR was performed as described [29].

### Full-length sequence and phylogenetic analysis of ChiVMV

The RT-PCR was performed using six degenerate primers for ChiVMV genome amplification (Additional file 1: Table S1). The 5′- and 3′-terminal sequences of ChiVMV were obtained through rapid amplification of cDNA ends (RACE) assays using SMARTer™ RACE cDNA Amplification kit (Clontech, Mountain View, USA) according to the manufacturer’s protocol. The obtained PCR products were cloned into pEASY-T1 Cloning Vector (Transgen Biotech) and sequenced (Sangon Biotech). The DNAMAN package (Version 8.0.8, Lynnon Biosoft, USA) was used for the full-length genome alignment. Amino acid sequence of ChiVMV-LZ was compared with those of other eleven ChiVMV isolates (GenBank Accession No. AGN92430.1, AGN92431.1, AGE47830.1, NP982308.1, AMV75279.1, ACX53640.1, ALH24936.1, CAB43195.2, CAP39938.1, ADP30806.1, ADP30807.1) obtained from GenBank. Four published pepper-infecting potyviruses (BAJ10980.1, ACE80681.1, CAJ57401.1 and ABG56784.1) were used as outgroup members. A total of sixteen sequences were aligned using the ClustalW algorithm of MEGA7 [30]. The phylogenetic tree of ChiVMV was constructed using 1000 bootstrap repeat sequences as matrices in the phylogenetic process of the adjacent connections [31].

### RT-RPA assay for ChiVMV detection

One pair of primers (RPA-ChiVMV-F: 5′-AAGATATGGGCTTCAAAGAAACCTTACCG-3′ and RPA-ChiVMV-R: 5′-CCTACCCTACCGTCCAGTCCGAACATCCT-3′) used for RT-RPA was designed based on the conserved coat protein genes for ChiVMV-LZ detection. The general guidelines for designing the RPA primers were followed in RPA manufacturers’ websites (http://www.twistdx.co.uk/en/support/rpa-assay-design-2), which recommended one pair of primers with 30–35 nucleotides for the optimal formation of recombinase/primer. To optimize the reaction time, RPA reactions were executed for 10, 20 and 30 min respectively using the cDNA from ChiVMV-infected tobacco plants. Conventional RT-RPA assays were performed in a 38 °C water bath for 20 min using a TwistAmp Basic kit (TwistDX, Cambridge, UK). The 50 μL reaction volume included 29.5 μL of rehydration buffer, 2.4 μL of each RPA-ChiVMV-F/R primer (10 μM), 2.5 μL of magnesium acetate (280 mM), 12.2 μL of nuclease-free water, and 1.0 μL of cDNA. RPA amplicons were purified using a SanPrep Column PCR Product Purification kit (Sangon Biotech) and then analyzed by 2% agarose gel electrophoresis. To evaluate the specificity of the established RT-RPA assay, ChiVMV-, TSWV-, potato virus Y (PVY)- or tobacco vein banding mosaic virus (TVBMV)-infected tobacco plants were tested, with the healthy tobacco plants as negative controls. For the sensitivity assessment, total RNA extracted from ChiVMV-infected tobacco plant was diluted to 1 ng, 10 pg, 1 pg, 100 fg, 10 fg and 1 fg, respectively. The diluted templates were then used for the first-strand cDNA synthesis with the M-MLV reverse transcriptase as instructed (Promega, Madison, USA). Each cDNA template was detected using RT-RPA and RT-PCR, respectively. To evaluate the feasibility of RT-RPA for ChiVMV detection in the field, a total of 71 tobacco plants with ChiVMV or viral-like symptoms were detected by RT-RPA and RT-PCR, respectively.

## Results

### Identification of ChiVMV infecting tobacco plants

**Fig. 1:**
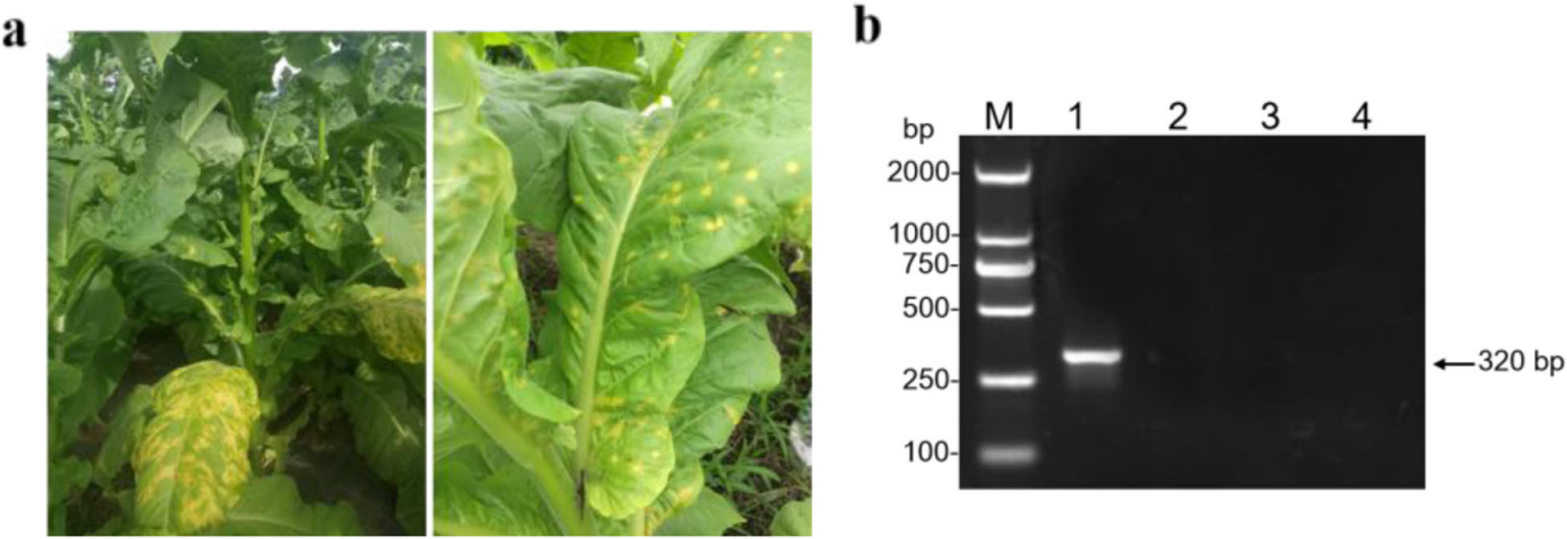
Identification of ChiVMV infecting tobacco plants. **a** Typical symptoms of chlorosis and round bright spots on the leaves of field-grown tobacco plants collected from Luzhou city, Sichuan province, China. **b** Viral detection in selected field-grown tobacco leaves using four universal primers by RT-PC*R. lane* M, *Trans*2K DNA marker; lane 1, primers PotyF/PotyR; lane 2, primers CMVCPf/CMVCPr; lane 3, primers Tob-Uni1/Tob-Uni2; lane 4, primers TSWVf/TSWVr

During the investigation of viral diseases in tobacco in Luzhou, Sichuan in Jun 2018, leaf samples with yellowing and shrinking symptoms were collected from field-grown tobacco (Fig. 1 a). Four pairs of universal primers (CMVCPf/CMVCPr, Tob-Uni1/Tob-Uni2, PotyF/PotyR and TSWVf/TSWVr) were used to detect the collected tobacco samples by RT-PCR, and an expected 320-bp fragment was obtained only with primers PotyF/PotyR (Fig. 1 b). After cloning and sequencing, the obtained nucleotide sequences of PCR products were aligned by BLASTn in NCBI database. The results showed that the sequences had over 98% homology with that of ChiVMV. Therefore, we preliminarily determined that the symptomatic tobacco samples were infected by ChiVMV, which was named ChiVMV-LZ.

### Pathogenicity of ChiVMV-LZ on different host plants

**Fig. 2:**
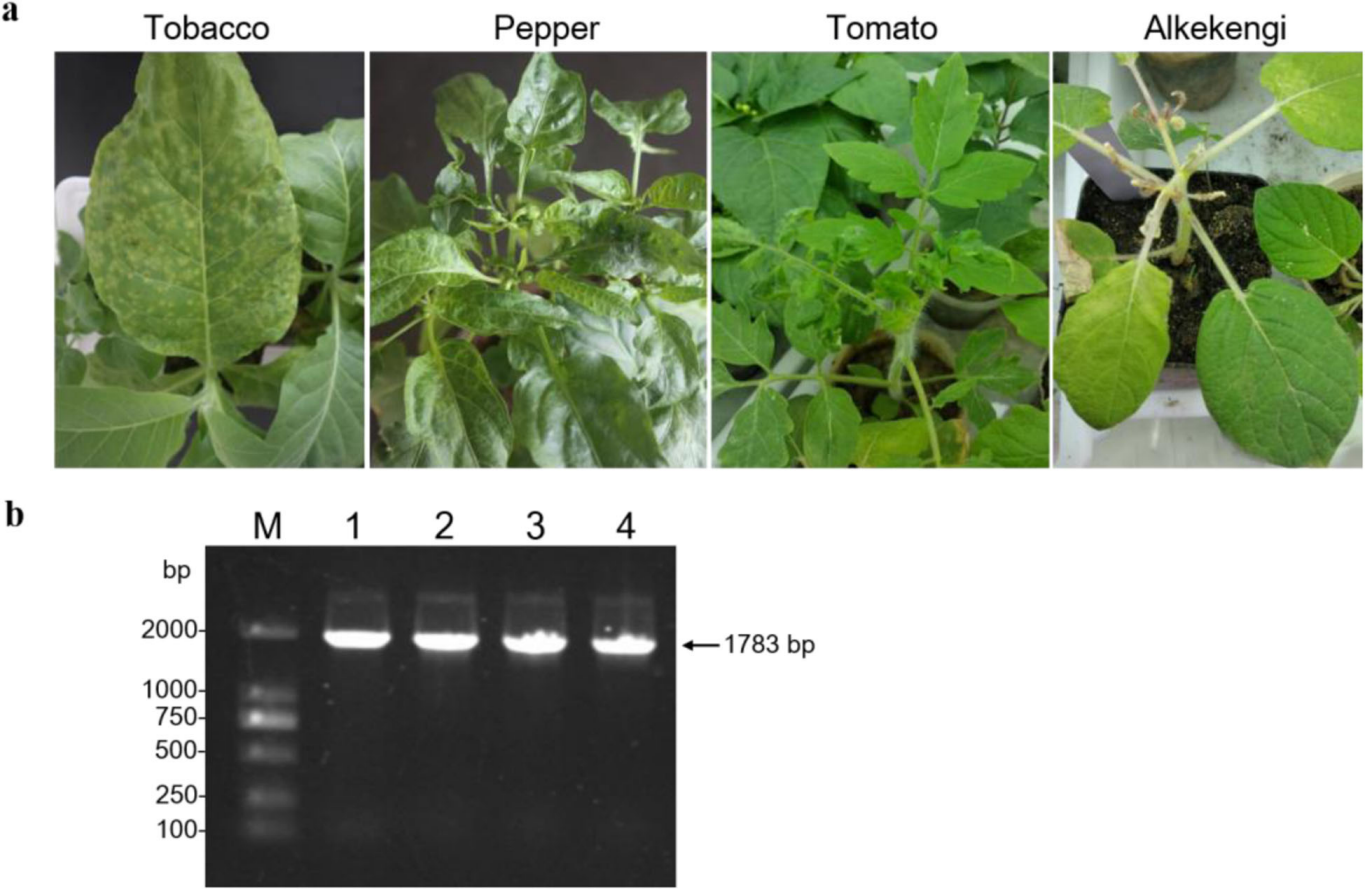
Symptom diversity of ChiVMV infection on different host plants. **a** Symptoms of ChiVMV-infected tobacco, pepper, tomato and alkekengi plants. The symptoms were observed at ten days post inoculation with ChiVMV. **b** RT-PCR detection of ChiVMV in different host plants using primers Chi6-F/*R. lane* M, *Trans*2K DNA marker; lane 1–4, ChiVMV-infected tobacco, pepper, tomato and alkekengi plants, respectively

To explore the pathogenicity of ChiVMV-LZ, the virus was mechanically inoculated onto different host plants. After ten days post inoculation (dpi), ChiVMV infection resulted in various symptoms in all tested solanaceous plants, including tobacco, pepper, tomato and alkekengi. Many round bright spots appeared on the leaves of ChiVMV-infected tobacco plants, with the manifestation of chlorosis and yellowing. Moreover, the leaves shrank with droopy and folded leaf margin, especially for the lower leaves. ChiVMV-infected pepper leaves prominently showed darker vein stripes, shrinkage of leaf margin and deformity of leaf surface, especially in young leaves. For ChiVMV-infected tomato plants, the upper leaves shrank, rolled up and stretched slowly. The alkekengi plants infected by ChiVMV showed crimped, yellowed, and almost completely unextended tender leaves (Fig. 2 a). With RT-PCR detection using the specific primers Chi6-F/R (Additional file 1: Table S1), all the tested host plants showed positive infection of ChiVMV (Fig. 2 b). These results demonstrated the pathogenicity of ChiVMV-LZ on different host plants.

### Full-length genomic sequence and phylogenetic analysis of ChiVMV

**Fig. 3:**
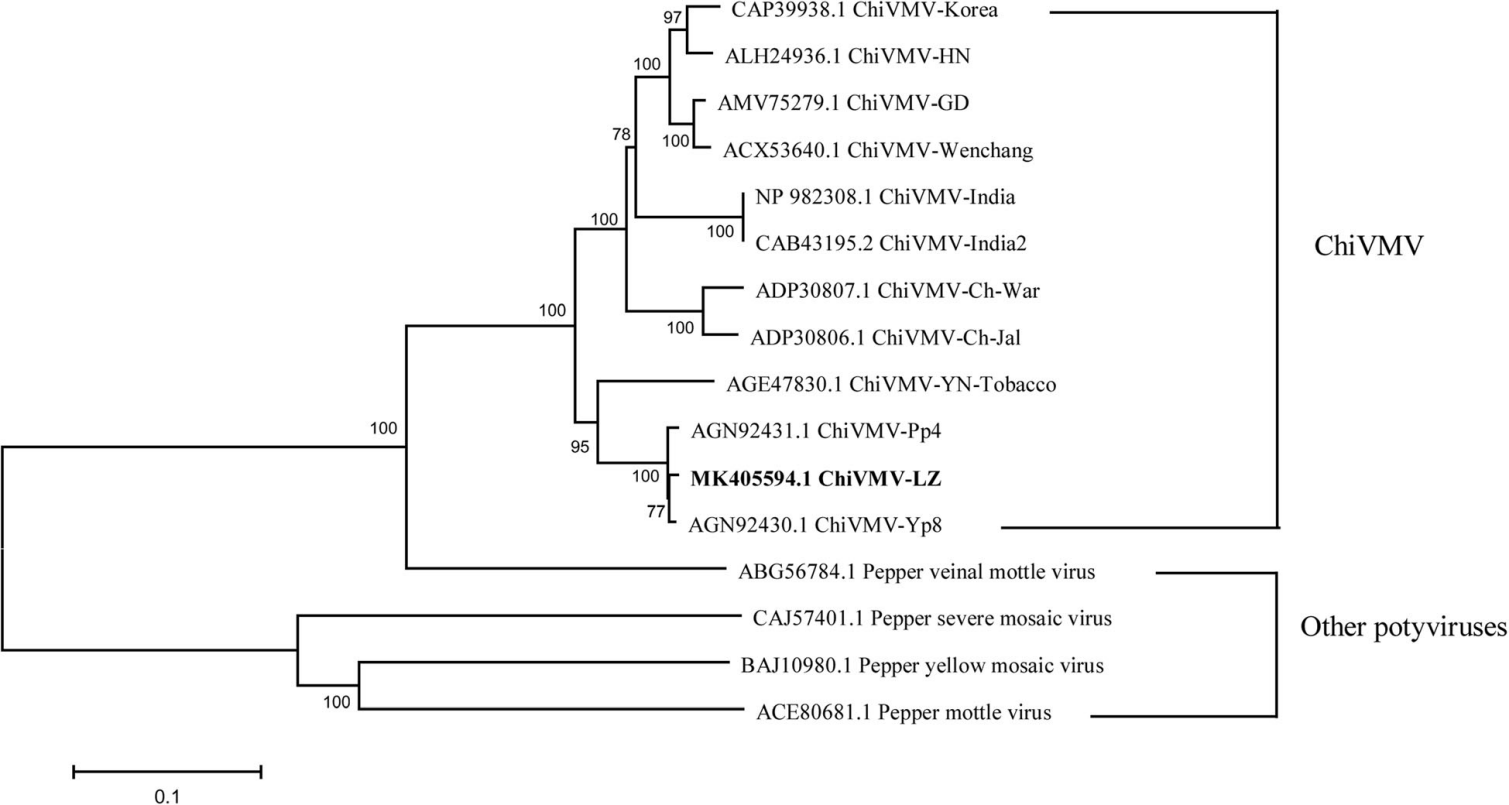
Phylogenetic analyses of ChiVMV and other potyviruses based on amino acid sequences. The phylogenetic trees were generated using the neighbor-joining method by MEGA7 software. The percentage of replicate trees in which the associated taxa clustered together in the bootstrap test (1000 replicates) was shown next to the branches

Near full-length genomic sequence of ChiVMV-LZ was obtained by RT-PCR using primers designed based on the sequences of reported ChiVMV isolates. Six sequenced fragments of 1944 bp, 1581 bp, 1728 bp, 1579 bp, 1871 bp and 1780 bp in length were obtained using primers Chi-F1/R1, Chi-F2/R2, Chi-F3/R3, Chi-F4/R4, Chi-F5/R5 and Chi-F6/R6, respectively (Additional file 3: Fig. S1; Additional file 1: Table S1). The 5′- and 3′-terminal genomic sequences of ChiVMV-LZ were determined by 5′ and 3′ RACE assays, respectively (Additional file 3: Fig. S1). The complete genomic sequence of ChiVMV-LZ (GenBank accession No. MK405594.1) was determined to be 9742 nt in length. The nucleotide homology of ChiVMV-LZ with other eleven ChiVMV isolates deposited in GenBank database was compared (Additional file 2: Table S2). The results showed that the nucleotide homology was 79.9~97.8% between ChiVMV-LZ and other eleven isolates. Notably, the nucleotide sequence identity value between ChiVMV-LZ and Sichuan isolates ChiVMV-Yp8 from pepper plants was the highest (97.8%). The identity values were 80.2~82.3% with the isolates from other provinces in China and 79.9~81.1% with the isolates from Korea and India (Additional file 2: Table S2). In addition, the sequence of ChiVMV-LZ was compared with the published sequences of potyviruses infecting pepper plants, including pepper vein mottle virus (PVMV), pepper mottle virus (PepMoV), pepper yellow mosaic virus (PepYMV) and pepper severe mosaic virus (PepSMV), with 49.0~64.7% nucleotide sequence identity (Additional file 2: Table S2). The amino acid-based neighbor-joining phylogenetic analysis showed that the isolates of ChiVMV from Sichuan province were clustered in one branch (Fig. 3), suggesting that ChiVMV-LZ had the closest relationship with the isolates of ChiVMV-Yp8 and ChiVMV-Pp4. Tobacco-infecting ChiVMV-LZ and pepper-infecting ChiVMV-Yp8 and -Pp4 probably came from the same strain, which were geographically related (Sichuan province). These results indicated that ChiVMV-LZ probably originated from infected pepper plants, and tobacco as a ChiVMV reservoir will probably increase the spread likelihood of ChiVMV to other solanaceous crops.

### Establishment of a RT-RPA assay for ChiVMV detection

**Fig. 4:**
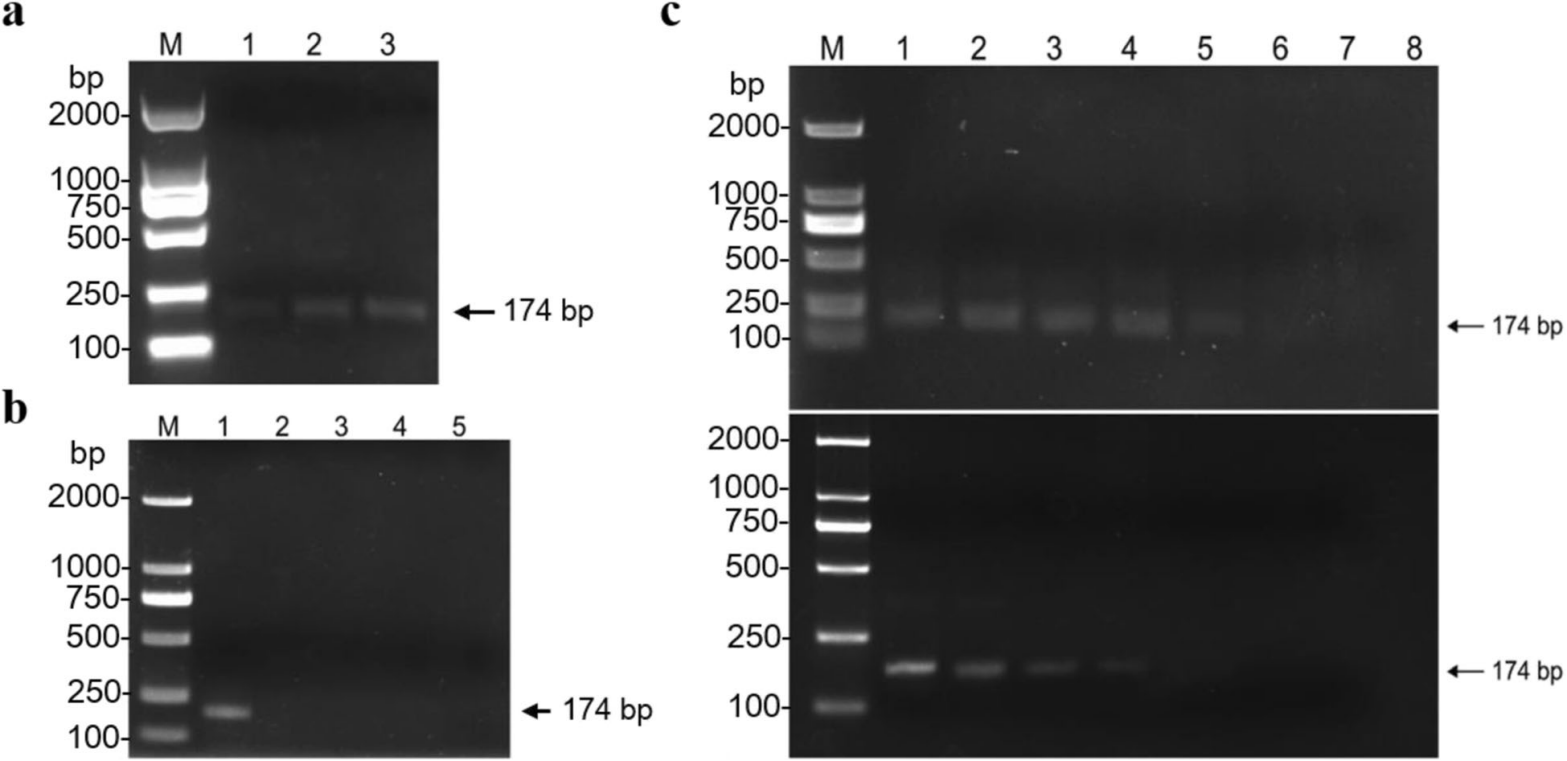
Establishment of RT-RPA assay for ChiVMV detection. **a** Optimization of RPA reaction time. Lane M, *Trans*2K DNA marker; lanes 1–3, DNA products amplified by RPA reaction for 10 min, 20 min or 30 min, respectively. **b** Specificity analysis of RT-RPA. Lane M, *Trans*2K DNA marker; lane 1, ChiVMV-infected tobacco plants; lane 2, PVY-infected tobacco plants; lane 3, TSWV-infected tobacco plants; lane 4, TVBMV-infected tobacco plants; lane 5, distilled water control. **c** Sensitivity comparison of RT-RPA and RT-PC*R. lane* M, *Trans*2K DNA marker; lane 1–6, a series of dilutions of total RNA extracted from ChiVMV-infected tobacco plants with concentrations of 1 ng, 10 pg, 1 pg, 100 fg, 10 fg and 1 fg, respectively; lane 7, distilled water; lane 8, healthy tobacco plants

In order to detect ChiVMV conveniently and quickly, a RT-RPA method was established. Based on the optimization of the reaction conditions of RT-RPA assay, 20 min were selected as the reaction time in this study (Fig. 4 a). To evaluate the specificity of the established RT-RPA assay, ChiVMV-, PVY-, TSWV- or TVBMV-infected tobacco plants were used. The results revealed that only ChiVMV-infected tobacco plants showed clear and positive band in the agarose gel electrophoresis assay (Fig. 4 b), indicating that the designed primers for RT-RPA assay were specific to detect ChiVMV. For sensitivity evaluation, a series of dilutions of total RNA (1.0 × 10^3^ ng/μL) extracted from ChiVMV-infected tobacco plants, including 1 ng, 10 pg, 1 pg, 100 fg, 10 fg and 1 fg, were prepared and amplified by RT-PCR and RT-RPA, respectively. The RT-RPA method could detect transcripts with RNA concentration of 10 fg, while RT-PCR could produce positive reaction with that of at least 100 fg (Fig. 4 c), suggesting that RT-RPA was approximately 10-fold more sensitive than RT-PCR based on the agarose gel electrophoresis assay. These results showed that the established RT-RPA was a reliable and rapid method for ChiVMV detection with high specificity and sensitivity.

### Application of RT-RPA assay for ChiVMV detection in field-collected tobacco sample

**Fig. 5:**
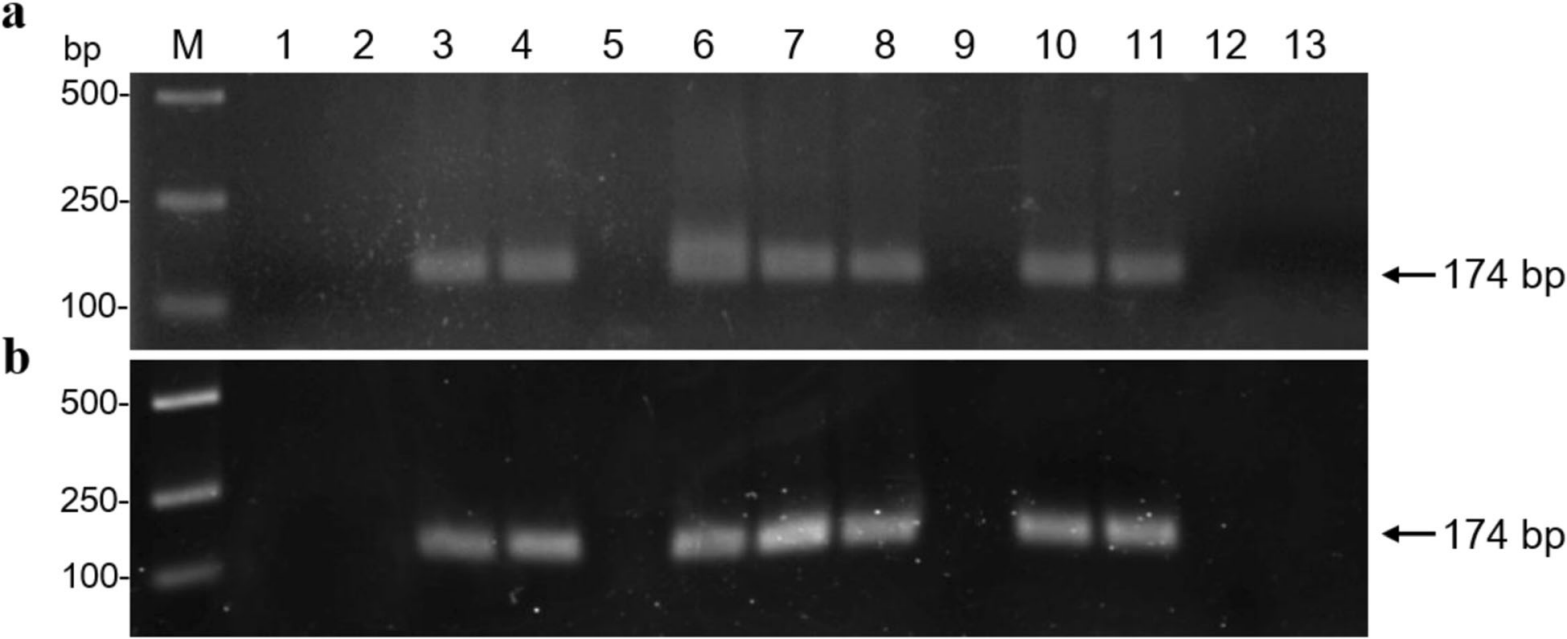
Detection of natural ChiVMV infection in selected field-collected tobacco leaves. **a** Using RT-RPA assay. **b** Using RT-PCR assay. Lane M, *Trans*2K DNA marker; lane 1, healthy tobacco leaves; lane 2, distilled water; lane 3, ChiVMV-infected tobacco plants; lanes 4–13, random field-collected tobacco samples with ChiVMV or viral-like symptoms

To evaluate the feasibility of RT-RPA method for ChiVMV detection in the field, 71 samples with ChiVMV or viral-like symptoms collected from Luzhou (Sichuan, China), Fuling (Chongqing, China) and Bijie (Guizhou, China) were tested by RT-RPA and conventional RT-PCR assays, respectively. The results showed that 56 samples showed ChiVMV-positive reaction by RT-RPA assay, and only 53 samples were positive by RT-PCR (Table 1). However, these three samples with negative ChiVMV in RT-PCR assay also developed typical symptoms soon, indicating that they were infected by ChiVMV but failed to be detected by RT-PCR assay. In addition, the ChiVMV-positive samples by RT-RPA assay showed clear bands in the agarose gel electrophoresis similar to that of RT-PCR (Fig. 5), suggesting its comparable performance to RT-PCR. These results demonstrated that the established RT-RPA assay was a rapid and sensitive technique for ChiVMV detection, and could be successfully applied in the field-collected samples.

**Table 1:** Comparative performance of RT- RPA and RT- PCR assays for ChiVMV detection in field-collected tobacco samples

Areas	RT-RPA		RT-PCR		Total
	Positive	Negative	Positive	Negative	
Luzhou	16	7	16	7	23
Bijie	19	5	18	6	24
Fuling	21	3	19	5	24
Total	56	15	53	18	71

## Discussion

Although with great efforts on different control strategies, plant viruses remain a great challenge and has caused huge economic losses in tobacco production every year in China [32]. The effectiveness of disease-controlling strategies may be affected by genetic diversity and variation in viral population composition [33]. Therefore, it is of great significance to timely identify and detect plant viruses. ChiVMV found in pepper, tomato and tobacco plants spreads rapidly in recent years, and its scope of damage has increasingly expanded [17, 34]. Sichuan province is one of the main tobacco-producing regions in China, with a large number of pepper plants scattered in the tobacco area. In this study, ChiVMV-LZ was isolated from the infected tobacco plants in Luzhou, southwest of Sichuan province, China (Fig. 1). The complete genomic sequence of ChiVMV-LZ was obtained and showed typical structural characteristics of *Potyvirues* [35]. The identity value analysis revealed that ChiVMV-LZ was most closely related to ChiVMV-Yp8 isolated from pepper plants in Sichuan, suggesting that ChiVMV-LZ might originate from infected pepper plants around tobacco plants. The CP genes of ChiVMV isolates from Fujian and Hunan provinces had the highest identity with those from Korea (> 95%), indicating that ChiVMV isolates from pepper plants in China had a tendency of genetic differentiation [36]. Similarly, ChiVMV-LZ had the highest nucleotide sequence identity (97.8%) with ChiVMV-Yp8 isolate from Sichuan, 82.3% homologous with ChiVMV-YN isolate from Yunnan, while the homology with these isolates from Hunan, Guangdong and Hainan provinces being only 80.2~80.8%, further indicating that ChiVMV had genetic differentiation and geographical differences. The homology of ChiVMV and other four viruses in the same genus was significantly lower than the classification criteria for species of *Potyvirus*, either at the nucleotide level (< 76%) or the amino acid level (< 82%), which also confirmed that ChiVMV was an independent specie of *Potyvirus*, belonging to cluster E [37].

ChiVMV has become one of the major viral pathogens affecting tobacco production in Sichuan. Since 2010, the detection rate of ChiVMV in suspected tobacco with viral diseases in Sichuan has exceeded 3% [18]. Of note, the damage scope by ChiVMV infection in China is currently expanding. The timely detection of pathogen is very important to predict, identify and prevent tobacco virus diseases. It is necessary to develop a rapid method to detect ChiVMV for early diagnosis and prevention of virus transmission. Previously, RT-PCR was the most widely used method to detect ChiVMV in infected plants and aphid tissues. However, there are several inherent shortcomings within RT-PCR method, such as requirement of special instruments, demanding and time-consuming. Previous studies also reported that RT-LAMP was 10 to 1000 times more sensitive than traditional RT-PCR because of its higher amplification efficiency [38, 39]. However, RT-LAMP method needs more pairs of primers and a relatively higher reaction temperature. Of note, the enzyme in the RPA reaction mixture could interact with the amplification products and inhibit DNA migration in gel. Therefore, the RPA amplicons must be purified and then separated by gel electrophoresis. In this study, a PCR purification kit with proven satisfying purification efficiency, was selected to purify the RT-RPA products to obtain better results. [26, 40].

Here is the very first study that the ChiVMV genome was amplified by RT-RPA. This method could yield a detectable signal with only one pair of primers, at a single temperature (38 °C), in 20 min for high specificity and sensitivity. As shown, the sensitivity of RT-RPA analysis was about 10 times higher than that of conventional RT-PCR based on the agarose gel electrophoresis. To determine the specificity of RT-RPA for ChiVMV detection, we selected other related viruses (i.e., PVY, TSWV and TVBMV) infecting tobacco as controls. No amplification products were observed except for ChiVMV-infected tobacco samples. The established RT-RPA assay was also successfully applied with field-collected samples, suggesting that at basic heating device (38 °C) could be used to effectively amplify the molecular target. As reported [41–43], the RT-RPA could proceed between 36 °C and 42 °C without affecting its efficiency and performance. A simple battery-powered portable instrument could be used to achieve accurate detection, thereby reducing the cost of the analysis [44, 45]. Previously, RPA has been successfully adapted to detect two potyviruses, yam mosaic virus (YMV) and yam mild mosaic virus (YMMV), which could be performed using crude sap extract from fresh or stored samples and RNA purification was not required [46]. Therefore, RPA would also be applied outside laboratory settings, even in low-resource fields. Taken together, thermal cycle instrument is not necessary within the RT-RPA method, which greatly simplifies the process and contributes to the on-site detection of ChiVMV in the field.

## Conclusions

In conclusion, we isolated and identified a new ChiVMV isolate from infected tobacco in Luzhou, Sichuan, China. ChiVMV-LZ was genetically related to these isolates from pepper plants in Sichuan province, indicating that ChiVMV-LZ might originate from pepper, which increased the probability of cross transmission of ChiVMV in Sichuan province. The phylogenetic sequence differentiation of ChiVMV isolates further enriched our understanding of the molecular and biological characteristics of ChiVMV. Meanwhile, we established an efficient RT-RPA method for rapid detection of ChiVMV with high sensitivity and specificity. The RT-RPA assay could provide rapid and efficient diagnosis of ChiVMV infection both in laboratory settings and low-resource fields.

## Supplementary information

**Additional file 1: Table S1.** Primers used in the article.

**Additional file 2: Table S2.** The genomic sequence identity between ChiVMV and other potyviruses infecting pepper plants.

**Additional file 3: Figure S1**. Schematic diagram of cloning strategy of ChiVMV full-length genome. The relative positions of primers and RT-PCR products were shown as line segments.

## Data Availability

All data generated or analyzed during this study are included in this published article.

## Abbreviations

ChiVMV: Chilli veinal mottle virus
ELISA: Enzyme-linked immunosorbent assay
MYSV: Melon yellow spot virus
ORF: Open reading frame
PepMoV: Pepper mottle virus
PepSMV: Pepper severe mosaic virus
PepYMV: Pepper yellow mosaic virus
PVMV: Pepper vein mottle virus
PVY: Potato virus Y
RACE: Rapid amplification of cDNA ends
RT-LAMP: Reverse transcription loop-mediated isothermal amplification
RT-PCR: Reverse transcription-polymerase chain reaction
RT-RPA: Reverse transcription-recombinase polymerase amplification
TVBMV: Tobacco vein banding mosaic virus
WSMoV: Watermelon silver mottle virus
YMMV: Yam mild mosaic virus
YMV: Yam mosaic virus
