# Supplementary material for: Characterization and a RT-RPA assay for rapid detection of Chilli Veinal mottle virus (ChiVMV) in tobacco
Source: Virol J. 2020 Mar 10;17:33. doi: 10.1186/s12985-020-01299-w (PMC7065361; doi:10.1186/s12985-020-01299-w)
Supplement: Supplementary file 1 — Additional file 1: Table S1. Primers used in the article. [file 12985_2020_1299_MOESM1_ESM.docx]

**Additional file 1:**

**Table S1. Primers used in the article.**

| **Primer** | **Sequence (5**′**-3**′**)^*^** |
| --- | --- |
| Chi1-F | CAAGCAWTCAAGCATTCAAGC |
| Chi1-R | CTGGTGAWCCATCATCATAAG |
| Chi2-F | GAGYTRATYCAGCCAACTGATG |
| Chi2-R | CATYTCTTGTTCYTCTATGTGC |
| Chi3-F | AATTTGTACAGGAGAAGATAG |
| Chi3-R | CTTCATTYGCTATDATTGTTGG |
| Chi4-F | TCAAYTATGGAGAGAGGATTC |
| Chi4-R | GTGGCTTGACCAGTCTGTCG |
| Chi5-F | CARGCATACTTTGTTAAAGAC |
| Chi5-R | TCYACBACTGTTGATGGTTGTC |
| Chi6-F | GCTGATGGRACAATAGTCAAG |
| Chi6-R | CATRAAYCATAACAATAGTCTTTG |
| PotyF | ATGGTHTGGTGYATHGARAAYGG |
| PotyR | TGCTGCKGCYTTCATYTG |
| CMVCPf | ATGGACAAATCTGAATCAACCA |
| CMVCPr | TCAGACTGGGAGCACCCCAGACGT |
| Tob-Uni1 | ATTTAAGTGGASGGAAAAVCACT |
| Tob-Uni2 | GTYGTTGATGAGTTCRTGGA |
| TSWVf | ACTTCAGACAGGATTGGAG |
| TSWVr | GCATTAGGATTGCTGGAGC |

* B=C/G/T; H= A/C/T; K= G/T; R=A/G; W=A/T; Y=C/T; S=C/G; V=A/C/G
