# Supplementary material for: Characterization and a RT-RPA assay for rapid detection of Chilli Veinal mottle virus (ChiVMV) in tobacco
Source: Virol J. 2020 Mar 10;17:33. doi: 10.1186/s12985-020-01299-w (PMC7065361; doi:10.1186/s12985-020-01299-w)
Supplement: Supplementary file 2 — Additional file 2: Table S2. The genomic sequence identity between ChiVMV and other potyviruses infecting pepper plants. [file 12985_2020_1299_MOESM2_ESM.docx]

**Additional file 2:**

**Table S2. The genomic sequence identity between ChiVMV and other potyviruses infecting pepper plants.**

| **Virus Abbreviation**_ **GenBank Number** | **Genome Size (nt)** | **Nucleotide Identity (%)** | **Amino Acid Similarity (%)** |
| --- | --- | --- | --- |
| ChiVMV-Yp8_KC711055 | 9741 | 97.8 | 97.6 |
| ChiVMV-Pp4_KC711056 | 9741 | 97.5 | 97.2 |
| ChiVMV-YN_JX088636 | 9739 | 82.3 | 88.4 |
| ChiVMV-WC_GQ981316 | 9717 | 80.1 | 86.9 |
| ChiVMV-HN_KR296797 | 9710 | 80.2 | 85.9 |
| ChiVMV-GD_KU987835 | 9721 | 80.8 | 87.1 |
| ChiVMV-Korea_AM909717 | 9710 | 80.3 | 85.1 |
| ChiVMV-Jal_GU170807 | 9705 | 81.1 | 85.8 |
| ChiVMV-War_GU170808 | 9702 | 80.9 | 85.3 |
| ChiVMV-India1_NC005778 | 9711 | 79.9 | 83.9 |
| ChiVMV-India2_AJ237843 | 9711 | 79.9 | 83.9 |
| PVMV_DQ645484 | 9792 | 64.7 | 70.2 |
| PepSMV_AM181350 | 9890 | 49.1 | 45.1 |
| PepYMV_AB541985 | 9745 | 49.2 | 44.9 |
| PepMoV_EU586124 | 9640 | 49.0 | 44.8 |
