# Supplementary material for: Characterization and a RT-RPA assay for rapid detection of Chilli Veinal mottle virus (ChiVMV) in tobacco
Source: Virol J. 2020 Mar 10;17:33. doi: 10.1186/s12985-020-01299-w (PMC7065361; doi:10.1186/s12985-020-01299-w)
Supplement: Supplementary file 3 — Additional file 3: Figure S1. Schematic diagram of cloning strategy of ChiVMV full-length genome. The relative positions of primers and RT-PCR products were shown as line segments. [file 12985_2020_1299_MOESM3_ESM.docx]

**Additional file 3:**


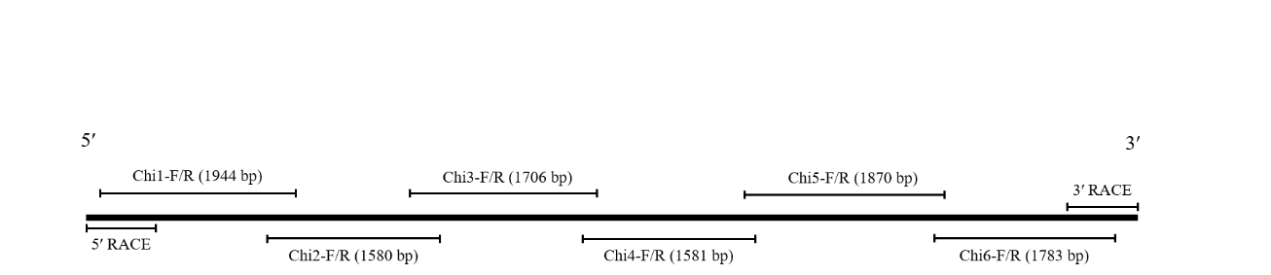


Figure S1. Schematic diagram of cloning strategy of ChiVMV full-length genome. The relative positions of primers and RT-PCR products were shown as line segments.
